# Metabolic response to extended continuous positive airway pressure in premature infants

**DOI:** 10.1038/s41390-025-04670-1

**Published:** 2025-12-22

**Authors:** Philip L. Ballard, Kelvin D. MacDonald, Julia Harris, Thaybeth I. Malave-Mendez, Mitzi Go, Alec Martin, Kristin Milner, Cheryl Chapin, Robert Tepper, Dara G. Torgerson, Cindy T. McEvoy

**Affiliations:** 1 Department of Pediatrics, University of California, San Francisco, CA USA; 2 Department of Pediatrics, University of Oregon Health and Science University, Portland, OR USA; 3 Pharmaceutical Sciences and Pharmacogenomics Graduate Program, University of California, San Francisco, CA USA; 4 Department of Pediatrics, University of Indiana School of Medicine, Indianapolis, IN USA; 5 Epidemiology and Biostatistics, University of California, San Francisco, CA USA

## Abstract

**Background:**

Extending the duration of continuous positive airway pressure (CPAP) in preterm infants with respiratory distress may improve lung function.

**Objective:**

Assess the impact of extended CPAP (eCPAP) on the metabolomic profile and relationship to lung function.

**Design/methods:**

Infants ≤32 weeks’ gestational age were randomized to bubble eCPAP with room air or discontinued CPAP (dCPAP). Functional residual capacity (FRC) was measured at randomization and at 2 weeks. Blood samples obtained during treatment were subjected to UHPLC:MS/MS (Metabolon Inc.) and metabolomic data were analyzed by ANCOVA.

**Results:**

eCPAP infants had greater increases in both FRC (2.1-fold, *p* = 0.005) and weight (1.13-fold, *p* = 0.03). Of 1230 total metabolites detected, levels of 87 were higher at *p* < 0.05 and 75 were lower in eCPAP infants. A new plateau level for key metabolites occurred at 5 ± 2 days after discontinuing CPAP. Sub pathway enrichment (*p* < 0.05) occurred for bilirubin degradation products (7.7-fold) and acylcarnitines (3.5-fold), all higher in dCPAP. Blood levels of some metabolites were correlated with FRC and weight gain.

**Conclusion:**

Discontinuation of CPAP altered the blood metabolome and impaired gain in FRC and weight. Extending CPAP may reduce the work of breathing, improve mitochondrial utilization of fatty acids, and provide more energy for lung and body growth.

**Impact:**

- Discontinuing CPAP in premature infants recovering from respiratory distress altered levels of 14% of detected blood metabolites within 1 week and reduced gain in lung function and body weight at 2 weeks.
- Biochemical sub pathway enrichment occurred for acylcarnitines with higher circulating levels of 22 fatty acid metabolites in infants coming off CPAP.
- Extending CPAP beyond the current guidelines for discontinuation may reduce the work of breathing, improve mitochondrial utilization of fatty acids, and provide more energy for lung development and body growth.

## Introduction

Preterm birth is the leading cause of respiratory morbidity in infancy and is linked to persistent respiratory conditions such as wheezing and asthma.^1,2^ Premature infants have reduced airway flow and parenchymal diffusion capacity compared to term infants,^3,4^ and these impairments often persist into adulthood and may predispose individuals to chronic obstructive pulmonary disease (COPD).^2,5–8^ Thus, strategies that enhance lung growth early in life may have long term benefit and reduce respiratory morbidity in adulthood.

Early application of nasal continuous positive airway pressure (CPAP), originally developed by Gregory et al.,^9^ is currently standard of care for management of preterm infants with respiratory distress.^10^ CPAP increases functional residual capacity (FRC) and improves gas exchange, reducing the need for invasive mechanical ventilation and associated lung injury.^11–13^ In experimental models, chronic CPAP increases lung volume, likely related in part to stenting airways and alveoli open, reducing the work of breathing, and stimulating stretch-induced alveolar growth.^14–17^

We previously reported that 2 weeks of extended room air CPAP (eCPAP) in stable premature infants recovering from respiratory distress, compared to discontinuation of CPAP (dCPAP) to room air, increased lung volumes at both 2 weeks and at discharge, suggesting that beneficial effects of CPAP may persist.^18^ In a subsequent randomized trial, we again evaluated the effect of an additional 2 weeks of CPAP in stable preterm infants and demonstrated improved lung function after 2 weeks of treatment (FRC) and also at 6 months of age (alveolar volumes, forced expiratory flows and lung diffusion capacity).^19^ The latter study serves as the parent clinical trial for the current sub-study investigating metabolic responses to eCPAP. We hypothesized that eCPAP in preterm infants would alter the blood metabolome and identify candidate biochemical pathways influenced by respiratory status.

Untargeted metabolomic analysis involves separation and identification of low molecular weight chemicals by ultrahigh performance liquid chromatography and mass spectrometry **(**UHPLC-MS/MS) to provide quantitative data on hundreds of metabolites in a biofluid. Previous studies have examined the metabolome in premature infants using tracheal aspirate/lung lavage fluid,^20–23^ blood,^24,25^ and urine,^26–28^ identifying putative biomarkers for bronchopulmonary dysplasia (BPD) or metabolic responses to treatments, however, there is no metabolome-wide information available for effects of ventilatory assistance in the premature infant. In the current study, we used untargeted metabolomic analysis of blood samples from a sub-set of premature infants in the recent eCPAP trial to identify metabolites with altered levels and to investigate associations with physiological parameters.

## Methods

### Study design

An investigator-initiated, single-center, single-blind RCT was conducted in the Oregon Health & Science University (OHSU) level IV neonatal intensive care unit (NICU), evaluating lung function at 6 months corrected age as the primary outcome (NCT#0429556). The protocol was approved by the OHSU Institutional Review Board and informed consent was obtained.

The parent trial design and methodology, including a CONSORT diagram, are described in detail in our recent publication.^19^ In brief, 276 infants >24 and ≤32 weeks of gestational age (GA) at birth were screened and 108 infants met study criteria and consented. 100 infants requiring CPAP for ≥24 h for clinical care, either for initial respiratory support or post-extubation and who met respiratory stability criteria for ≥12 hours at <35 weeks postmenstrual age (PMA), were enrolled. Infants were randomized to 2 weeks of extended CPAP (eCPAP, *n* = 54) or discontinuation of CPAP to room air (dCPAP, *n* = 46) after they met CPAP respiratory stability criteria.^18,29,30^ All infants received enteral feeds with fortified donor breast milk during the study period. Because continuing nasal prongs in the dCPAP group was not ethically or logistically feasible, treatment blinding was not possible. The clinical team, outcome assessors, and statistician, however, were blinded to allocation.

As previously published, clinical care at OHSU continues premature infants on CPAP until “CPAP respiratory stability criteria”^18,30^ are met for ≥12 h at which time CPAP is discontinued and the infant is placed on room air. CPAP was administered as bubble CPAP with distending pressure (Infant Bubble CPAP system, Fisher & Paykel, Irvine, CA) through appropriately sized Hudson nasal prongs (Teleflex, Morrisville, NC) with chinstrap applied and an 8 French orogastric tube. The nares were assessed every 3–4 h and prongs repositioned as needed.

A non-sedated measurement of FRC by nitrogen washout was done in the NICU on study day 0 and again on day 14 when eCPAP was discontinued.^31–33^ Infants were also studied at ~6 months age as an outpatient after sedation with oral chloral hydrate. Measurements of alveolar volume and lung diffusion capacity were obtained using an induced respiratory pause technique at an elevated lung volume (30 cm H_2_O).^4,34,35^ Forced expiratory flow measurements in triplicate were obtained using the raised volume rapid thoracic compression (RVRTC) technique as described.^34,36^

### Metabolomic analyses

A single convenience blood sample was collected from infants during the 14-day study period with a total of 64 available samples (33 eCPAP and 31 dCPAP infants). Of the total of 100 infants enrolled in the parent study, blood samples were not available on 36 infants because parents did not consent for blood draw (a separate opt in/out portion of the consent), infants failed their randomized treatment assignment to the dCPAP or eCPAP group, inadequate blood volume was obtained, or infants were transferred before a convenience clinical blood draw was obtained. Blood samples were stored at −70 °C for 1–4 years before transfer to Metabolon Inc. (Morrisville, NC) for untargeted metabolomic analysis by UHPLC-MS/MS in a single batch that included a set of internal standards to adjust as needed for instrument variability.^25,27,28^

The metabolite dataset comprised a total of 1366 compounds of both known identity (named by Metabolon as of April 2025, 82%) and unnamed (18%) chemicals. Area under the peak was obtained for each biochemical, and the data presented are expressed relative to the median value of all samples for each chemical. We limited analysis of metabolite levels between eCPAP and dCPAP infants to those metabolites that had detectable levels in ≥70% of samples in at least one group of infants. Additionally, we eliminated caffeine and 9 caffeine derivatives from analysis because levels of all were markedly elevated in infants on caffeine at the time of the blood sample (73% of eCPAP and 52% of dCPAP infants, *p* = 0.08) compared to infants not receiving caffeine. A total of 1230 metabolites were further analyzed.

### Statistical analysis

Statistical analyses were performed on log transformed data using ArrayStudio and Jupyter Notebook. Metabolite levels between treatment groups were analyzed by analysis of covariance (ANCOVA) with adjustment for postnatal age at time of sample and significance presented as p values. An estimate of the false discovery rate (*q*-value) was also calculated to take into account the multiple comparisons that normally occur in metabolomic-based studies. We used volcano plotting for a visual overview of the metabolomic data, dimensionality reduction using Principal Component Analyses (PCA) in R, and Partial Least Squares Discriminant Analysis (PLS-DA) using Metabolon’s bioinformatics portal. For PCA, a scree plot of ascending PCs versus % of the variance indicated the point of maximum curvature (elbow) at PC 2/3 (data not shown); accordingly, we limited PCA visualization to PC1 vs PC2. For PLS-DA, we performed 5-fold cross-validation to obtain an estimate of model performance and evaluate overfitting. We used linear regression to examine the direct associations of metabolite levels with postnatal age, study days, FRC, and weight. Univariate comparisons between outcomes and metabolite abundance were analyzed using Fisher’s exact test.

Metabolite set enrichment analysis was performed to identify biochemical sub-pathways enriched for metabolites that vary between eCPAP and dCPAP infants. Metabolites of known identity from Metabolon (as of April 2025) were annotated to 86 sub-pathways with ≥3 detected metabolites. Metabolite set enrichment tests were performed for each sub-pathway. The number of expected metabolites differing between groups at *p* < 0.05 in each sub-pathway was calculated as the fraction of total differential metabolites (0.139) x number of metabolites in each sub-pathway. This value (rounded to the nearest integer) was compared to the observed proportion of differential metabolites in each sub-pathway using a Fisher Exact Test.

## Results

### Infant characteristics

Infants in the two groups were similar in GA (mean 30 weeks PMA), birthweight ( ~ 1500 g), racial/ethnic and sex distribution, and morbidities. Similarly, there were no significant differences in postnatal age at randomization or collection of blood samples (Table 1, upper). The only medications infants received during the study period were caffeine (63.5%), Vitamin D3 (60.9%), ferrous sulfate (60.9%) and bifidobacterium infantis (probiotic, 45%), with no significant differences (*p* < 0.05) in exposure rate between eCPAP and dCPAP infants.

**Table 1:** Demographic and physiological parameters of infants.

	eCPAP	dCPAP	eCPAP/dCPAP	
	(*n* = 33)	(*n* = 31)	fold	p
**Demographics**				
Gestational Age (wk)	29.8/1.7	30.2/2.0	0.99	0.34
Birth Weight (g)	1458/318	1501/341	0.97	0.60
White/Black/Hispanic/Asian	20/0/6/3	15/3/4/2		0.34
Sex (m/f)	22/11	15/16		0.21
Age Randomization (days)	13.9/12.4	13.1/15.8	1.06	0.28
Age Sample Collected (days)	24.7/13.1	22.9/16.3	1.08	0.62
Interval Randomize/Sample (days)	10.8/2.7	9.8/5.1	1.10	0.31
Antibiotic Treatment	5	1		0.20
Bronchopulmonary dysplasia	1	2		NS
**Physiological**				
FRC day 0 (ml)	42.2/10	44.3/10.1	0.95	0.95
FRC day 14 (ml)	55.8/11.5	49.8/8.1	1.12	**0.02**
FRC change day 0-14 (ml)	13.7/9.0	5.6/10.5	2.45	**0.002**
FRC change day 0-14 (%)	35.5/25.7	16.9/25.7	2.10	**0.006**
Weight day 0 (g)	1561/237	1595/253	0.98	0.58
Weight change day 0-14 (%)	31.8/7.1	28.2/6.0	1.13	**0.031**
Caloric intake day 0-14 (kcal/kg/day)	126.7/9.8	127.3/8.6	1.02	0.19
Lung diffusion capacity at 7 mo (ml/min/mmHg)	3.53/0.94	2.59/0.94	1.36	**0.0002**
Alveolar volume at 7 mo (ml)	521/145	387/145	1.35	**0.0007**

Mean/SD.

Statistics by Student’s unpaired t test, Mann Whitney test or Fisher exact test.

*p* values < 0.05 in **bold**.

*FRC* functional residual capacity.

Mean values for FRC performed on day zero of the study were identical in eCPAP and dCPAP infants, however, the increase in FRC at day 14 was 2-fold greater for eCPAP infants. Body weights at study entry were also similar in the 2 groups, but at day 14 eCPAP infants had gained 13% more weight (*p* = 0.03) with no difference in caloric intake. In pulmonary function studies at ~7 months, eCPAP infants had greater lung diffusion capacity (36%) and alveolar volume (35%) (Table 1, lower). These demographic and physiologic results for the subpopulation of infants with metabolomic studies are representative of data for the entire cohort in the trial (*n* = 100),^19^ and similarly, there were no significant differences between infants with and without metabolomic analyses.

**Fig. 1:**
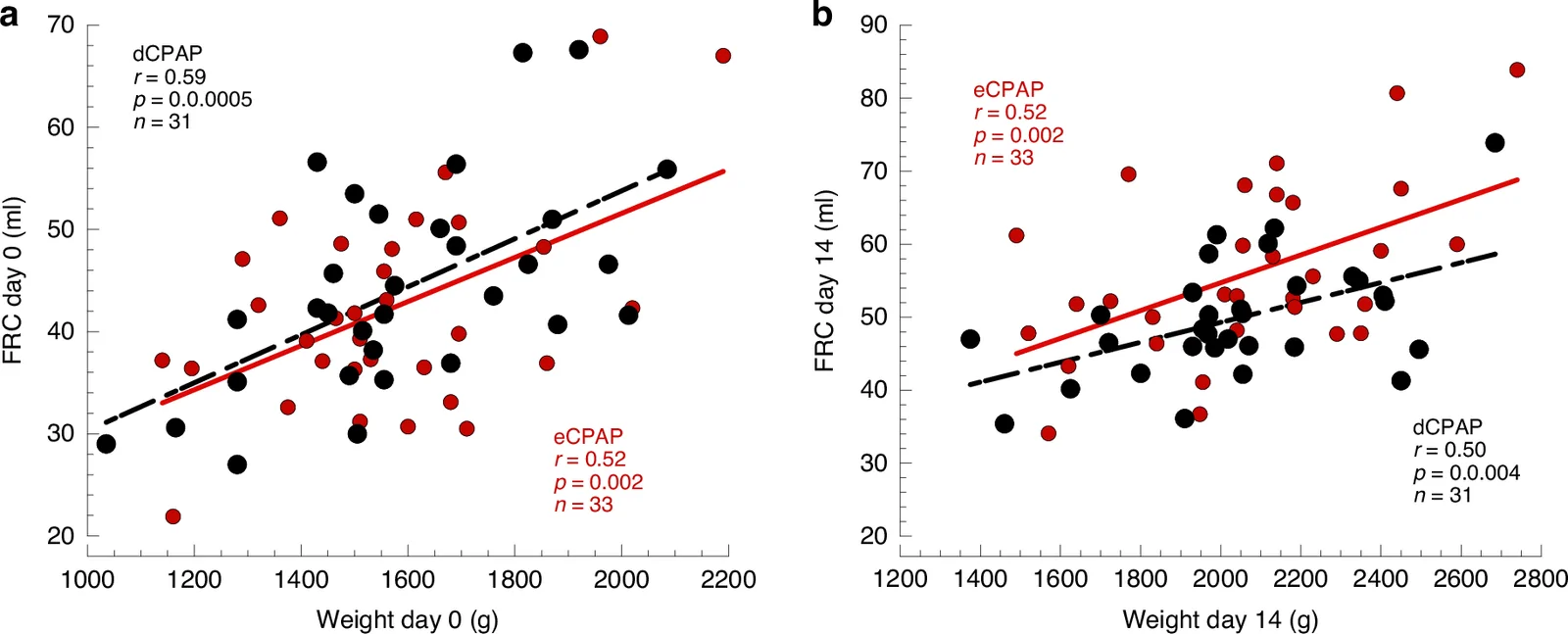
Association between body weight and FRC by linear regression. **a** infants on day 0 of study; **b** infants on day 14 of study. Distribution of values for eCPAP and dCPAP infants is similar at day 0; at day 14 FRC levels are generally higher for eCPAP vs dCPAP infants. Values for correlation coefficient (r), p of slope, and number of infants are shown within each figure.

As shown in Fig.1, FRC was correlated with body weight at both the beginning of the study (*r* = 0.55, *p* < 0.0001, *n* = 64) and at day 14 (eCPAP *r* = 0.52, *p* = 0.002, *n* = 33; dCPAP *r* = 0.50, *p* = 0.004, *n* = 31). FRC values were generally higher for eCPAP infants throughout the weight range at day 14 but not at day 0.

### Levels of blood metabolites

Overall, a total of 1230 different metabolites were detected that met quality control conditions; the identity of 980 was known by Metabolon. Regression analyses revealed a significant association between metabolite levels and postnatal age at collection of blood samples (range 6–79 days) for 38.2% of detected metabolites.

**Fig. 2:**
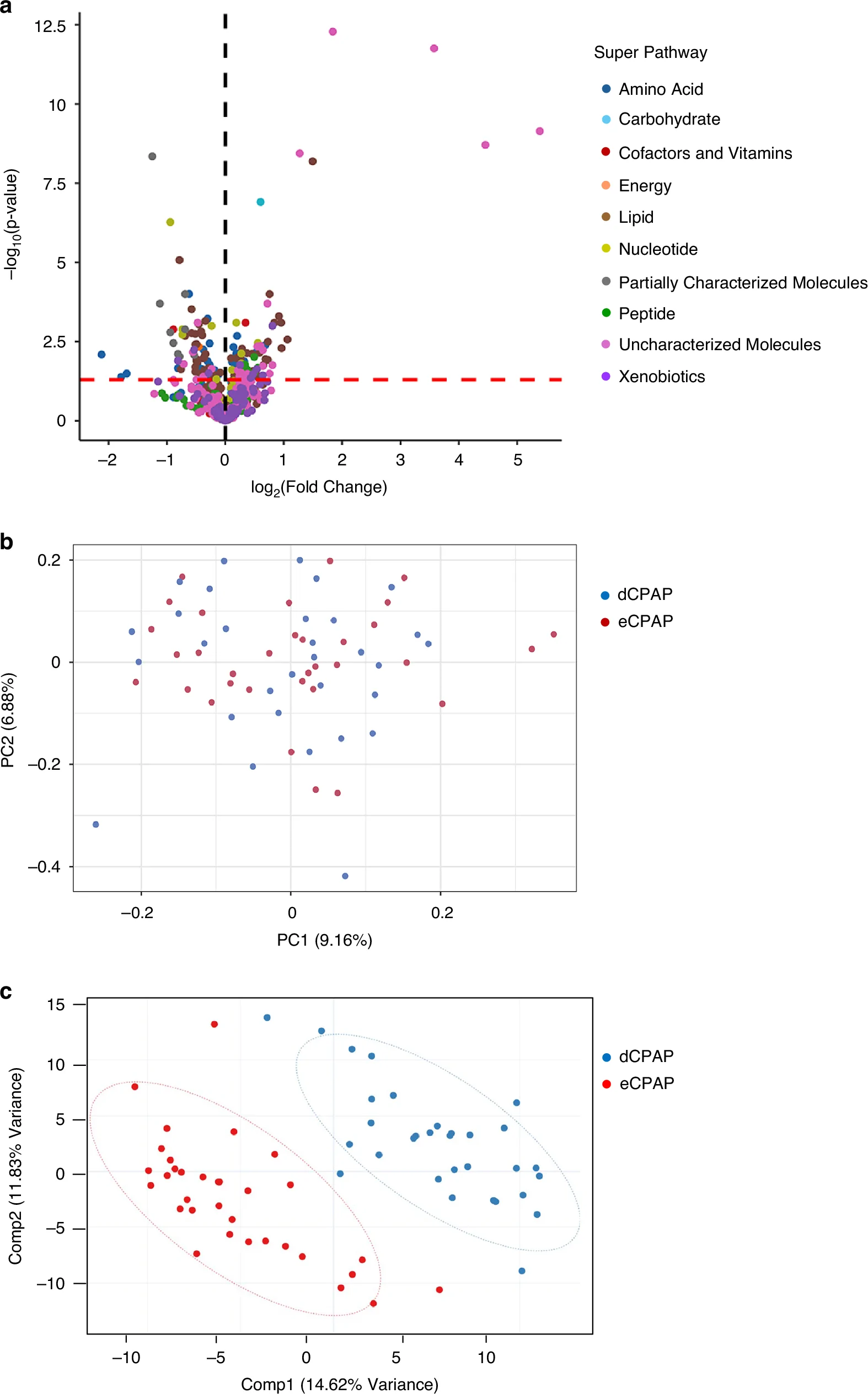
Scatter plots of metabolomic data. **a** Volcano plot of fold change and p value for metabolite levels in eCPAP vs dCPAP infants. Data are for 1230 metabolites color-coded by super pathway. Levels of 87 metabolites were higher (right of black dashed line) at p < 0.05 (above red dashed line) in eCPAP and levels of 75 were lower. **b** PCA plot showing no apparent clustering by eCPAP vs dCPAP, indicating no confounding related to overall variation in the data. **c** PLS-DA plot showing separation of eCPAP and dCPAP infants based on metabolic features that maximize the separation between groups. There were no outlier infants in this analysis. Dashed lines are 95% confidence levels. By 5-fold cross-validation analysis, the R2 value was 0.97 and the Q2 value was 0.48 on a scale of 0–1.0, indicating no overfitting and providing an estimate of predictive ability of the model.

Comparing metabolite data for eCPAP vs dCPAP infants, levels of 162 chemicals (13.2%) were different at *p* < 0.05 (differential metabolites) by analysis of covariance, adjusting for the effect of postnatal age at sample collection. The volcano plot illustrates the range of fold differences and of *p* values for metabolites, with higher levels in eCPAP infants for 87 differential metabolites and lower levels for 75 (Fig. 2a). Fold difference for these metabolites ranged from 1.06 to 41.9 (median 1.33), and *p* values from 0.05 to <10^−8^, and they represented all 10 biochemical super pathways (color coding in Fig. 2a) as identified by Metabolon, with highest counts for lipids (59) and amino acids (31). There was a similar number of metabolites per super pathway for those with higher vs lower levels with the following exceptions: 20 of 24 Unidentified chemicals (listed as X-#####) and 13 of 17 Peptide biochemicals were among metabolites with higher levels in eCPAP infants, and 9 of 9 Partially Characterized chemicals were among metabolites with lower levels.

By PCA scatterplot of PC1 vs PC2 (Fig. 2b), there was no apparent clustering by eCPAP vs dCPAP values, indicating no confounding related to overall variation in the data. Percent values for the variance explained by each principal component were 9.2% and 6.9%, respectively. PLS-DA (Fig. 2c) demonstrated separation of infants by treatment group with no outliers detected.

Table 2 lists the top 20 named, differential metabolites by *p* value separated by higher and lower values for eCPAP infants. Among metabolites with elevated levels, histidylphenylalanine, a product of protein degradation and 2-aminoheptanoate (homonorleucine), a 7-carbon alpha-amino acid, and glucuronate, which facilitates excretion of molecules, had the highest significance (*p* ≤ 10^−7^). For the 10 molecules with lower levels in eCPAP infants, there were 3 bilirubin degradation products and 3 acylcarnitines with p ≤ 0.003. Not shown in Table 2 are 6 Unnamed metabolites with *p* values < 10^−8^ and fold differences from 2.4- to 42-fold. The full list of differential metabolites is presented in Supplemental Table S1.

**Table 2:** Top 20 named metabolites with difference in levels between eCPAP and dCPAP infants.

Biochemical Name	Super Pathway	Sub Pathway	eCPAP/dCPAP			Detected
			fold	p	q	%
**Higher IN eCPAP**						
histidylphenylalanine	Peptide	Dipeptide	13.75	**<0.00001**	**<0.00001**	63
2-aminoheptanoate	Lipid	FA, Amino	2.82	**<0.00001**	**<0.00001**	100
Glucuronate	Carbohydrate	Aminosugar Metabolism	1.52	**<0.00001**	**0.00001**	100
Pregnenediol disulfate	Lipid	Pregnenolone Steroids	1.79	**0.0008**	**0.0345**	100
Nicotinamide riboside	Cofactors/Vitamins	Nicotinamide Metabolism	1.27	**0.0008**	**0.0345**	100
N1-methyladenosine	Nucleotide	Purine Metabolism, adenine	1.14	**0.0008**	**0.0345**	100
N-acetyltheanine	Xenobiotics	Food Component/Plant	1.75	**0.001**	**0.0365**	96
Creatinine	Amino Acid	Creatine Metabolism	1.15	**0.0021**	0.0561	100
Glycochenodeoxycholate 3-sulfate	Lipid	Primary Bile Acid Metabolism	2.09	**0.0027**	0.0693	100
Glycolithocholate sulfate	Lipid	Secondary Bile Acid Metabolism	1.66	**0.0032**	0.0814	70
**Lower IN eCPAP**						
Bilirubin degrade product C16H18N2O5(3)	Partially Characterized	Partially Characterized	0.42	**<0.00001**	**<0.00001**	73
Dihydroorotate	Nucleotide	Pyrimidine Metabolism, Orotate	0.52	**<0.00001**	**<0.00001**	100
Propionylcarnitine(C3)	Lipid	FA Metabolism (Acylcarnitine)	0.58	**<0.00001**	**0.0007**	100
Spermidine	Amino Acid	Polyamine Metabolism	0.65	**<0.00001**	**0.0081**	100
Bilirubin degrade product C16H18N2O5(1)	Partially Characterized	Partially Characterized	0.62	**0.0001**	**0.0093**	76
Bilirubin degrade product C17H18N2O4(1)	Partially Characterized	Partially Characterized	0.46	**0.0002**	**0.0114**	100
Myristoylcarnitine(C14)	Lipid	FA Metabolism (Acylcarnitine)	0.67	**0.0003**	**0.015**	100
Creatine	Amino Acid	Creatine Metabolism	0.81	**0.0006**	**0.0325**	100
Deoxycarnitine	Lipid	Carnitine Metabolism	0.79	**0.0007**	**0.0345**	100
Dihomo-linoleoylcarnitine	Lipid	FA Metabolism (Acylcarnitine)	0.73	**0.003**	**0.0365**	100

Metabolites in each category ordered by *p* and *q* value (ANCOVA).

% Detected, percent of all samples with level above lower limit of detection; *p* values < 0.05 in **bold**.

*FA* fatty acid.

The 1230 detected metabolites represented 117 sub-pathways, with 86 containing at least 3 metabolites. We examined whether differential metabolites were enriched in any sub-pathways by determining the observed number of differential metabolites compared to the expected number in each sub-pathway (Table 3). Enrichment occurred for bilirubin degradation metabolites and acylcarnitines (all acylcarnitines and the sub-category polyunsaturated fatty acids), with lower levels for all in eCPAP infants. By carbon chain length, long chain fatty acids represented 58% of the 22 differential acylcarnitine metabolites, with 25% for short chain and 8% for medium chain.

**Table 3:** Differential metabolites enriched by sub-pathway category.

Sub-Pathway	Metabolites with *p* < 0.05/total		Fold enrichment		eCPAP Levels	
	Observed	Expected	Observed/		Higher	Lower
			Expected	*p*		
Bilirubin synthesis/degradation	10/10	1.4/10	7.1	**0.0001**	0	10
FA metabolism (all acyl carnitines)	22/46	6.3/46	3.5	**0.0005**	0	22
FA metabolism (acyl carnitine, polyunsaturated)	7/8	1.1/8	6.4	**0.011**	0	7
FA metabolism (acyl carnitine, hydroxy)	5/8	1.1/8	4.5	0.12	0	5
FA metabolism (acyl carnitine, monounsaturated)	5/11	1.5/11	3.3	0.15	0	5

Enriched sub-pathways from a total of 86 sub-pathways with ≥3 metabolites.

Expected value is fraction of total metabolites differing at *p* < 0.05 (0.139) x number of metabolites in sub-pathway.

Data were rounded to the nearest integer for calculation of *p* value (Fisher exact test).

*p* values < 0.05 in **bold**.

Among the differential metabolites, an effect of postnatal age at sample collection on levels was notable for 5 sub-pathways: there was an age effect for 6 of 8 dipeptides with all decreasing; all 9 bilirubin degradation products decreased with age; all 3 primary bile metabolites increased with age; and 3 of 5 pyrimidine metabolites increased and 1 decreased. Of particular interest, for 22 differential acylcarnitine metabolites, 13 had a significant correlation with age at sample collection with levels of all increasing for both eCPAP and dCPAP infants; examples of postnatal age dependency for levels of propionylcarnitine (a 3-carbon fatty acid) and docosapentaenoylcarnitine (a 22-carbon polyunsaturated fatty acid) are shown in Supplemental Fig. S1. This age-related increase may reflect increasing intestinal maturity and more efficient absorption of fatty acids.^37^ The influence of postnatal age for all differential metabolites is shown in Supplemental Table S1.

**Fig. 3:**
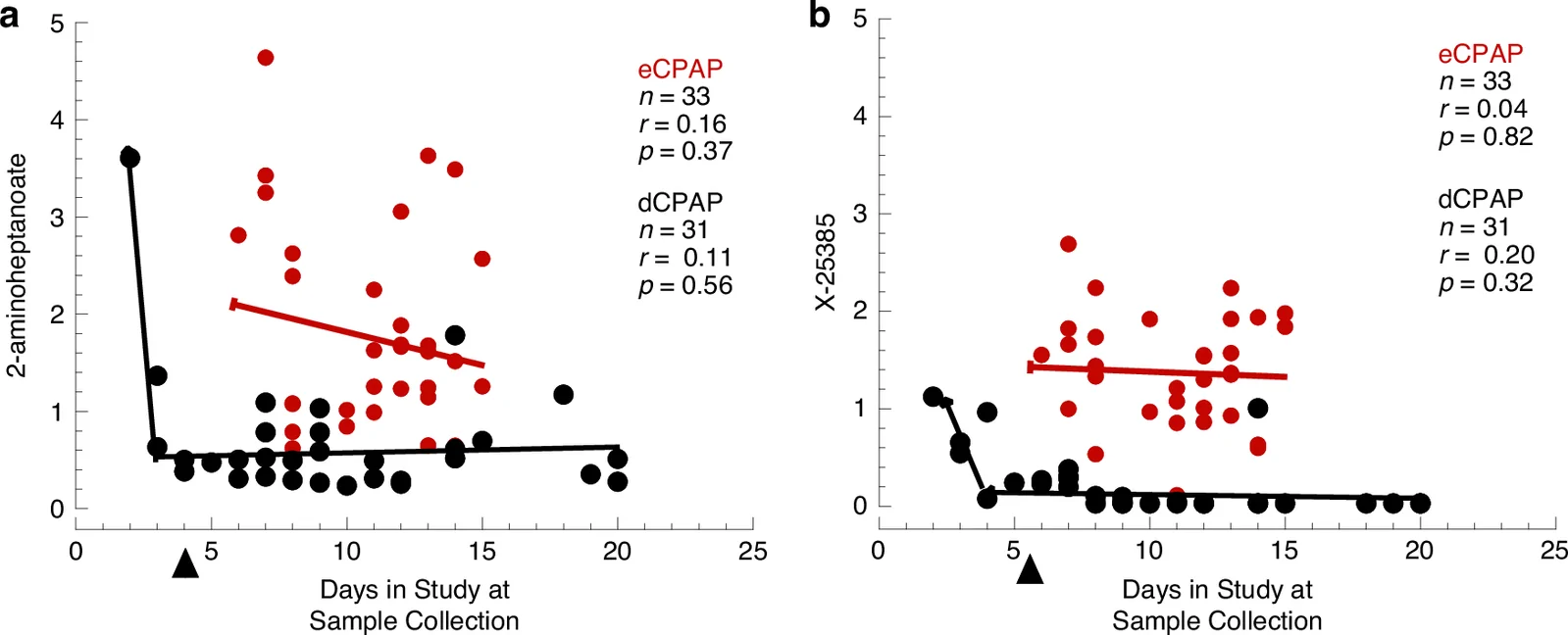
Time course of levels for 2 differential metabolites in eCPAP and dCPAP infants. Plateau levels in the dCPAP group (black symbols) are achieved at approximately 4 and 5 days (arrowheads) for 2-aminoheptanoate (**a**) and X-25385 (**b**), respectively, with no significant slope by linear regression for plateau values in both dCPAP and eCPAP (red symbols) infants.

Convenience blood samples were obtained between 2 and 20 days after study entry, based on the need for a blood draw for clinical indications (mean ~10 days for each group, Table 1). This study design allowed us to examine metabolite levels by time after discontinuing CPAP. For this cross-sectional analysis we selected the 28 differential metabolites with *p* < 0.01 eCPAP vs dCPAP and with no effect of postnatal age on levels. Time course results for 2 metabolites with higher levels in eCPAP infants illustrate decreasing levels in dCPAP infants over day 2–5 with plateau values thereafter. As expected, levels over time for infants continuing CPAP did not show a time-related effect (Fig. 3a and b). For all 28 metabolites, the time to plateau levels on discontinuing CPAP was 5.2 ± 1.7, range 2–10 days. Thus, the full metabolic effect of stopping CPAP in this cohort of infants is observed within a week.

### Relationship of metabolite levels to physiological effects

**Fig. 4:**
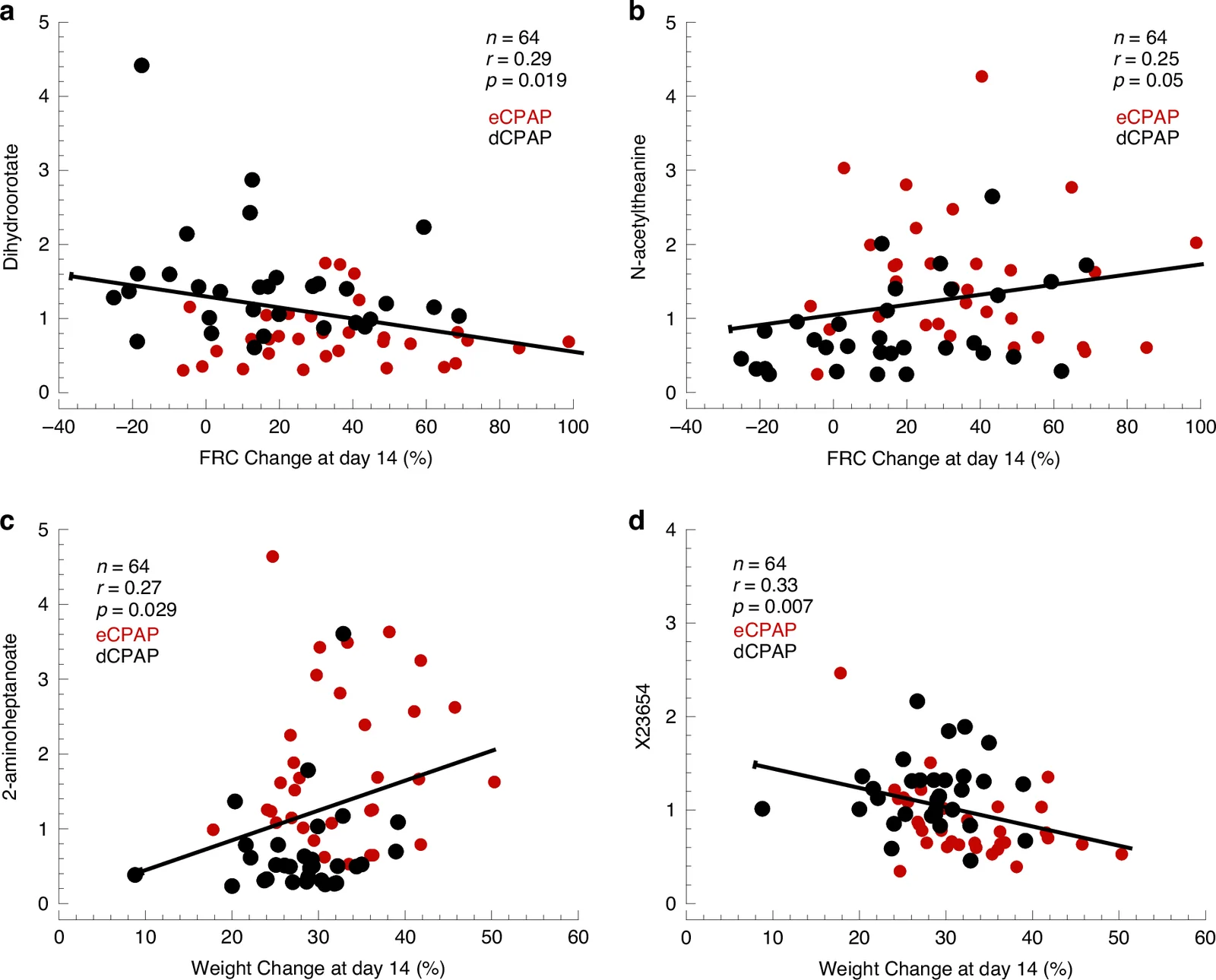
Relationship by linear regression between change in FRC and in weight gain vs levels of four differential metabolites. **a** dihydroorotate vs change in FRC. **b** N-acetyltheanine vs change in FRC. Linear regression analysis of data for 64 infants is shown. The direction of the slopes is consistent with lower and higher levels in eCPAP infants (red symbols) for dihydroorotate and N-acetyltheanine, respectively, and eCPAP values are shifted toward higher FRC in eCPAP infants. **c** 2-aminoheptanoate vs weight gain. **d** X23654 vs weight gain. The direction of the slopes is consistent with higher and lower levels in eCPAP infants (red symbols) for 2-aminoheptanoate and X23654, respectively, and eCPAP values are shifted toward higher weight gain in eCPAP infants.

We examined the relationship between levels of the 28 highly differential metabolites (*p* < 0.01) noted above and percent changes in FRC and body weight at study day 14 vs day 0. In linear regression analysis of all 64 infants, levels of 12 metabolites (43%) correlated (slope *p* < 0.1) with FRC; the slope was in the expected direction for each metabolite as shown in Fig. 4a and b for dihydroorotate and N-acetyltheanine vs FRC, respectively. In regression analysis for change in weight, 9 of the 12 metabolites showed a significant correlation, which was also in the expected direction for each metabolite (Fig. 4c, d). FRC and weight data as related to levels of these metabolites are summarized in Table 4. The similar findings for FRC and weight are consistent with an observed modest positive correlation between change in FRC and change in weight during the study period: *r* = 0.24, *p* = 0.057, *n* = 64.

**Table 4:** Metabolite levels and change in FRC and weight.

Metabolite		Linear Regression Analysis: Metabolite Levels				
	eCPAP/ dCPAP	vs % change FRC			vs % change weight	
	fold	*r*	*p* slope	Direction	*r*	*p* slope
histidylphenylalanine	13.75	0.39	**0.001**	Inc	0.25	**0.048**
2-aminoheptanoate	2.82	0.35	**0.0046**	Inc	0.28	**0.025**
N-acetyltheanine	1.75	0.25	**0.05**	Inc	0.08	0.53
X-24414	31.62	0.41	**0.0008**	Inc	0.21	**0.09**
X-25385	11.92	0.38	**0.003**	Inc	0.33	**0.007**
X-24571	41.91	0.24	**0.038**	Inc	0.25	**0.046**
X-24575	21.92	0.36	**0.064**	Inc	0.36	**0.009**
1-palmitoyl-2-linoleoyl-GPI	1.29	0.27	**0.03**	Inc	0.21	**0.09**
Gamma-carboxyglutamate	1.14	0.22	**0.085**	Inc	0.11	0.4
Dihydroorotate	0.52	0.29	**0.019**	Dec	0.15	0.21
X-23654	0.72	0.29	**0.02**	Dec	0.33	**0.007**
Isobutyrylcarnitine	0.78	0.31	**0.012**	Dec	0.24	**0.058**

*p* values < 0.05 in **bold**.

*Inc* increased, *Dec* decreased.

## Discussion

In this report, we describe metabolomic changes related to CPAP in premature infants with early respiratory distress. As hypothesized, discontinuing CPAP altered levels of ~14% of detected blood metabolites. Analysis by time for highly differential metabolites indicated that systemic metabolism is reset within a week of discontinuing CPAP. Levels of a subset of these differential metabolites were associated with the degree of improvement in lung FRC and body weight over 14 days in eCPAP infants. The findings suggest a role for altered metabolism and systemic bioenergetic status in the improvement of lung function and body weight observed with eCPAP treatment. Distinct metabolomic profiles have been described for various lung diseases,^38^ however, to our knowledge, this is the first study to report metabolic alterations associated with positive pressure respiratory support.

In the parent clinical trial for this investigation, and in a previous pilot study, we found that extending CPAP for 2 weeks in premature infants improved pulmonary function parameters at both 2 weeks and 7 months, suggesting a longer-term benefit.^18,19^ In adults, a limited number of studies have found improved pulmonary function with CPAP treatment. CPAP treatment (30 min daily) in adults with congestive heart failure was associated with progressive improvement in FEV1 and FVC over 2 weeks.^39^ Studies of nightly CPAP reported improved FEV1 and total lung capacity in patients with obstructive sleep apnea and COPD.^40^ These and other observations support the concept that CPAP improves alveolar expansion and stability and lessens work of breathing in various lung disorders.

FRC in premature infants is highly correlated with body weight.^41^ We also observed this relationship in our cohort at both study entry and finish, suggesting a close relationship between nutrition, weight, and lung function. We are not aware of previous reports of nutritional/growth benefits of CPAP in premature infants, although this topic has been examined in infants and adults with obstructive sleep apnea. In infants with airway malacia, CPAP treatment was associated with catch-up, long-term increases in weight and height percentiles.^42^ In adults, Siopi et al.^43^ reported a time dependent decrease in basal metabolic rate after patients began CPAP therapy, and Tachikawa et al.^44^ found an increase in body weight consistent with decreased energy expenditure. These responses are consistent with proposed effects of eCPAP to reduce work of breathing and presumably basal metabolic rate, providing increased energy for growth of lung parenchyma and body weight gain in premature infants recovering from respiratory distress.

Among named metabolites, higher fold differences for eCPAP vs dCPAP infants occurred for the dipeptide histidylphenylalanine (13.8-fold) and the alpha amino acid 2-aminoheptanoate (2-aminoheptanoic acid, homonorleucine, 2.8-fold). There is limited information on histidylphenylalanine beyond being a product of protein degradation. 2-aminoheptanoate is present in cord blood, infant urine, adult serum, and in adult cerebrospinal fluid where it is a biomarker for germ cell tumors,^28,45,46^ however, there is no information regarding its physiological function. Among named metabolites with lower levels in eCPAP infants, dihydroorotate had the greatest fold difference (1.9-fold lower, *p* < 10^−6^) with its metabolite orotate also being lower (1.7-fold, *p* < 0.001). Dihydroorotate is a substrate for the enzyme dihydroorotate dehydrogenase, which is linked to the respiratory complex of mitochondria; this reaction produces orotate, which is a precursor for de novo synthesis of pyrimidines that are essential for growth.^47^ Several metabolites not yet identified had even higher fold differences (3.6- to 31.6-fold) and were related to FRC. Some of these highly differential biochemicals are potential candidates as biomarkers for systemic responses to ventilatory strategies.

Adequate levels of exogenous fatty acids, in particular polyunsaturated fatty acids, are essential in premature infants for neurodevelopment and anti-inflammatory actions, and altered levels have been reported for infants developing BPD.^48^ In our study, levels of 22 acylcarnitines were higher in infants off CPAP, and this sub-pathway was enriched >3-fold over expected. This finding is consistent with findings in other disorders. Plasma acylcarnitines are elevated in inherited metabolic disorders involving defects in fatty acid oxidation.^49^ In addition, acylcarnitines were elevated in a group of patients with inherited mitochondrial myopathy, and levels correlated with both disease severity and reduced activity of respiratory chain complexes.^50^ In a metabolomic study of secondary carnitine deficiency in children with environmental enteric dysfunction, levels of acylcarnitines and other fatty acids were elevated.^51^ Acylcarnitines were among blood metabolites with higher levels in adult nonsurvivors vs survivors with acute respiratory failure/sepsis.^52^ Similar results for acylcarnitines as a marker of disease severity are reported for COVID-19.^53–56^ Elshenawy et al.^57^ performed a metabolomic study of placentas in preterm birth (with labor) compared to normal term delivery. Among differential metabolites, 17 of 41 acylcarnitines were higher in preterm placentas, which were exposed to inflammation and oxidative stress, than in term placentas; in addition, in vitro studies with placental homogenates found decreased fatty acid oxidation in preterm tissue. Considered together, these various findings suggest that levels of circulating acylcarnitines increase with severity of systemic disorders secondary to mitochondrial injury and dysfunction that reduces beta-oxidation and ATP production, resulting in reduced uptake and increased levels of circulating fatty acids. In the case of discontinuing CPAP in premature infants, mitochondrial dysfunction could arise from intermittent hypoxemia secondary to apneic episodes plus increased level of systemic inflammation related to increased work of breathing.^41^ These effects could promote increased levels of reactive oxygen/nitrogen species and oxidative damage to mitochondrial lipids, proteins and nucleic acids and reduce energy production.^58^ Future studies are needed to examine the role of energy metabolism in CPAP therapy and to determine whether selected acylcarnitines may be potential biomarkers for the status of mitochondrial and bioenergetic function and body/lung growth in infants.

Levels of all 10 detected degradation metabolites of bilirubin were lower in eCPAP infants. These closely related compounds are produced primarily in gut bacteria.^59^ This observation could reflect different levels of circulating bilirubin between groups; however, we could not evaluate this possibility as bilirubin was below the level of detection in all blood samples. It is also possible that the gut microbiota differs in quantity or diversity between infants on and off CPAP, perhaps related to nutritional status. Intake of the probiotic bifidobacterium infantis, which influences microbiota diversity and may promote infant growth,^60,61^ was similar in the two groups (45 and 52% for eCPAP and dCPAP, respectively) and thus likely did not influence bilirubin metabolism or levels of the 5 additional differential metabolites, 3-indoxyl sulfate, glycocholenate sulfate, glycodeoxycholate 3-sulfate, glycholithocholate sulfate, and 4-hydroxyhippurate, representing aromatic amino acid, bile metabolism and xenobiotic metabolism, respectively, that are reported to be primarily products of gut bacterial metabolism.^62^ CPAP can be associated with abdominal distension (CPAP belly), which can interfere with feeding, but no instances of distension were noted during the study and caloric intake was similar in each group. Understanding the possible impact of eCPAP on gut maturity and microbiota will require additional studies.

Intermittent hypoxemic episodes (IH) are common in premature infants and are associated with reduced weight gain, retinopathy of prematurity, necrotizing enterocolitis, bronchopulmonary dysplasia, and adverse neurodevelopmental outcome.^63–66^ Based primarily on animal data, adverse effects of hypoxemia in infants are proposed to involve increased inflammation and prooxidant status, particularly in mitochondria.^63,67–70^ Of interest, recent preliminary analysis of the parent eCPAP study found significantly fewer episodes of hypoxemia for eCPAP infants over the 14 days.^71^ Similarly, Yazdi et al.^72^ reported decreased intermittent hypoxemia on extended CPAP compared to low flow nasal cannulae. These observations suggest a possible role for reduced inflammation and oxidative stress in response to eCPAP; however, our study does not provide any data related to this proposal.

**Fig. 5:**
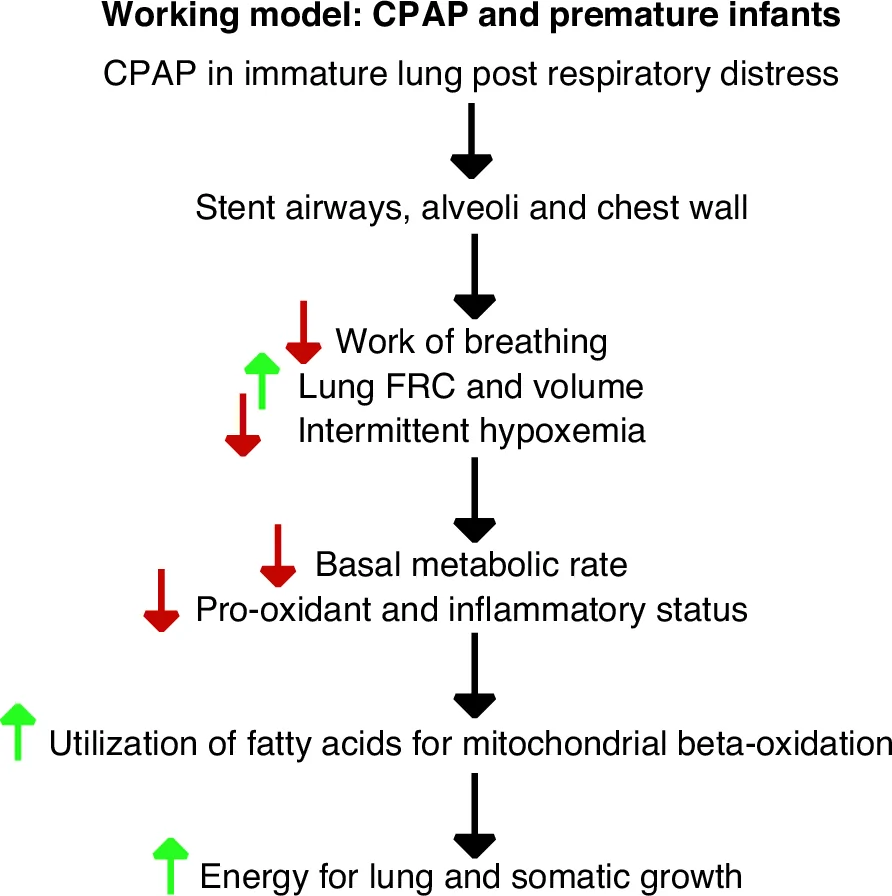
Proposed model for observed effects of eCPAP. The proposed responses to CPAP with green arrows are supported by results from the current study; other steps in the pathway are supported by data in the literature as discussed in the text.

Based on results of the eCPAP studies plus published data, we propose a hypothetical model for physiological events related to CPAP as outlined in Fig. 5. CPAP therapy of premature infants with immature lungs stents airways, alveoli and respiratory muscles and thus reduces the work of breathing and basal metabolic rate, increases FRC, and reduces intermittent hypoxemia and proposed systemic hypoxic/oxidative stress and inflammation.^11–14^ This improves mitochondrial function, including utilization of fatty acids for energy production via beta-oxidation.^63,67–70^ The improved bioenergetics provide energy for lung growth and maturation and for somatic growth.

There are methodological limitations to our metabolomic study. The number of infants in each group is relatively small and it will be important to examine metabolomic and weight changes associated with CPAP in additional studies with larger cohorts. Multiple uncontrolled confounders could contribute to the observed metabolomic differences. For example, it is known that the metabolomic profile of premature infants is strongly influenced by nutritional composition^28^; however, all infants in both groups received the same milk formulation (fortified donor breast milk) during the study period. Metabolite data are for blood and may not reflect levels in lung fluid, which was not available for these non-intubated infants. We used p < 0.05 for presentation of results between eCPAP and dCPAP infants, and corresponding q values for some metabolites were >0.1. While a higher *q*-value indicates diminished confidence, it does not necessarily rule out the significance of a result that may include significance in another dimension of the study, inclusion in a common pathway with highly significant compounds, or residing in a similar functional biochemical family with other significant compounds. It is important to note that our results do not establish causality, which will require additional longitudinal data related to metabolic rate, caloric balance, gut microbiota and levels of metabolites by targeted analysis.

The results from this study and those of the two associated RCTs indicate clinical benefits of eCPAP for 2 weeks in premature infants with no apparent adverse effects of eCPAP. However, there are limitations to these studies that preclude recommending extended CPAP treatment as routine clinical care for premature infants. The studies involved a relatively small number of infants ( ≤ 100 per study) from a single medical center, few extremely premature infants were included, and there was a predominance of maternally identified Caucasian race infants. Clinical and ethical considerations did not allow the use of nasal tubes in dCPAP infants and thus blinding of caregivers, although evaluators and the statistician were blinded to treatment group. Additional RCTs are needed to confirm the benefit/risk findings of extended CPAP beyond current respiratory stability criteria and to define optimal treatment parameters related to gestational age, postnatal age, severity of initial lung disease, and duration of extended CPAP; there is also a need for additional infant growth measurements, longer-term growth/development, and later pulmonary health/function.

In summary, we report that an extended time on CPAP in premature infants recovering from respiratory distress is associated with alterations of the blood metabolome that correlate with improved FRC and weight gain after 2 weeks. Our findings support a longer duration of CPAP in infants recovering from respiratory distress; however, further studies are needed to confirm benefit/safety and to define optimal treatment parameters.

## Supplementary information

Supplemental Figure S1

Supplemental Table S1

## Data Availability

The datasets generated and analyzed during the current study are available from the corresponding author on reasonable request.

## References

[1] Stocks, J., Hislop, A. & Sonnappa, S. Early lung development: lifelong effect on respiratory health and disease. *Lancet Respir. Med.* **1**, 728–742 (2013). 10.1016/S2213-2600(13)70118-8 24429276

[2] Jordan, B. K. & McEvoy, C. T. Trajectories of lung function in infants and children: setting a course for lifelong lung health. *Pediatrics* **146**, e20200417 (2020). 10.1542/peds.2020-0417 PMC7546086 32938776

[3] Friedrich, L. et al. Growth rate of lung function in healthy preterm infants. *Am. J. Respir. Crit. Care Med.* **176**, 1269–1273 (2007). 10.1164/rccm.200703-476OC PMC2176107 17885265

[4] Balinotti, J. E. et al. Growth of lung parenchyma in infants and toddlers with chronic lung disease of infancy. *Am. J. Respir. Crit. Care Med.* **181**, 1093–1097 (2010). 10.1164/rccm.200908-1190OC PMC2874450 20133928

[5] Stern, D. A., Morgan, W. J., Wright, A. L., Guerra, S. & Martinez, F. D. Poor airway function in early infancy and lung function by age 22 years: a non-selective longitudinal cohort study. *Lancet* **370**, 758–764 (2007). 10.1016/S0140-6736(07)61379-8 PMC2831283 17765525

[6] Guerra, S. et al. Fetal Origins of Asthma: A Longitudinal Study from Birth to Age 36 Years. *Am. J. Respir. Crit. Care Med.* **202**, 1646–1655 (2020). 10.1164/rccm.202001-0194OC PMC7737573 32649838

[7] Agusti, A. & Faner, R. Lung function trajectories in health and disease. *Lancet Respir. Med.* **7**, 358–364 (2019). 10.1016/S2213-2600(18)30529-0 30765254

[8] Bui, D. S. et al. Childhood lung function predicts adult chronic obstructive pulmonary disease and asthma-chronic obstructive pulmonary disease overlap syndrome. *Am. J. Respir. Crit. Care Med.* **196**, 39–46 (2017). 10.1164/rccm.201606-1272OC 28146643

[9] Gregory, G. A., Kitterman, J. A., Phibbs, R. H., Tooley, W. H. & Hamilton, W. K. Treatment of the idiopathic respiratory-distress syndrome with continuous positive airway pressure. *N. Engl. J. Med.* **284**, 1333–1340 (1971). 10.1056/NEJM197106172842401 4930602

[10] Subramaniam, P., Ho, J. J. & Davis, P. G. Prophylactic or very early initiation of continuous positive airway pressure (CPAP) for preterm infants. *Cochrane Database Syst. Rev.* **10**, CD001243 (2021). 10.1002/14651858.CD001243.pub4 PMC8521644 34661278

[11] Dunn, M. S. et al. Randomized trial comparing 3 approaches to the initial respiratory management of preterm neonates. *Pediatrics* **128**, e1069–e1076 (2011). 10.1542/peds.2010-3848 22025591

[12] Newman, N. S. et al. Early CPAP versus surfactant in extremely preterm infants. *N. Engl. J. Med.* **362**, 1970–1979 (2010). 10.1056/NEJMoa0911783 PMC3071534 20472939

[13] Morley, C. J. et al. Nasal CPAP or intubation at birth for very preterm infants. *N. Engl. J. Med.* **358**, 700–708 (2008). 10.1056/NEJMoa072788 18272893

[14] Xue, Z., Zhang, L., Liu, Y., Gunst, S. J. & Tepper, R. S. Chronic inflation of ferret lungs with CPAP reduces airway smooth muscle contractility in vivo and in vitro. *J. Appl. Physiol.* **104**, 610–615 (2008). 10.1152/japplphysiol.00241.2007 18096756

[15] Zhang, S., Garbutt, V. & McBride, J. T. Strain-induced growth of the immature lung. *J. Appl. Physiol.* **81**, 1471–1476 (1996). 10.1152/jappl.1996.81.4.1471 8904555

[16] Nguyen, T. M. et al. Stretch increases alveolar type 1 cell number in fetal lungs through ROCK-Yap/Taz pathway. *Am. J. Physiol. Lung Cell Mol. Physiol.* **321**, L814–L826 (2021). 10.1152/ajplung.00484.2020 34431413

[17] Edwards, Y. S. Stretch stimulation: its effects on alveolar type II cell function in the lung. *Comp. Biochem Physiol. A Mol. Integr. Physiol.* **129**, 245–260 (2001). 10.1016/s1095-6433(01)00321-x 11369549

[18] Lam, R. et al. The effect of extended continuous positive airway pressure on changes in lung volumes in stable premature infants: a randomized controlled trial. *J. Pediatr.* **217**, 66–72 e61 (2020). 10.1016/j.jpeds.2019.07.074 PMC7986570 31519441

[19] McEvoy, C. et al. Extended continuous positive airway pressure in preterm infants increases lung growth at 6 months. *Am. J. Respir. Crit. Care Med.* In press 10.1164/rccm.202411-2169OC (2025). PMC12005030 39977011

[20] Clish, C. B. Metabolomics: an emerging but powerful tool for precision medicine. *Cold Spring Harb. Mol. Case Stud.* **1**, a000588 (2015). 10.1101/mcs.a000588 PMC4850886 27148576

[21] Lal, C. V. et al. Early airway microbial metagenomic and metabolomic signatures are associated with development of severe bronchopulmonary dysplasia. *Am. J. Physiol. Lung Cell Mol. Physiol.* **315**, L810–L815 (2018). 10.1152/ajplung.00085.2018 PMC6295508 30113227

[22] Fabiano, A. et al. Metabolomic analysis of bronchoalveolar lavage fluid in preterm infants complicated by respiratory distress syndrome: preliminary results. *J. Matern Fetal Neonatal Med.* **24**(Suppl 2), 55–58 (2011). 10.3109/14767058.2011.606977 21781003

[23] Piersigilli, F. et al. Identification of new biomarkers of bronchopulmonary dysplasia using metabolomics. *Metabolomics* **15**, 20-32 (2019). 10.1007/s11306-019-1482-9 30830433

[24] La Frano, M. R. et al. Umbilical cord blood metabolomics reveal distinct signatures of dyslipidemia prior to bronchopulmonary dysplasia and pulmonary hypertension. *Am. J. Physiol. Lung Cell Mol. Physiol.* **315**, L870–L881 (2018). 10.1152/ajplung.00283.2017 PMC6295510 30113229

[25] Ballard, P. L. et al. Blood metabolomics in infants enrolled in a dose escalation pilot trial of budesonide in surfactant. *Pediatr. Res.* **90**, 784–794 (2021). 10.1038/s41390-020-01343-z PMC7814527 33469180

[26] Pintus, M. C. et al. Urinary (1)H-NMR metabolomics in the first week of life can anticipate BPD diagnosis. *Oxid. Med. Cell Longev.* **2018**, 7620671 (2018). 10.1155/2018/7620671 PMC6046120 30050661

[27] Torgerson, D. et al. The hydrocortisone-responsive urinary metabolome of premature infants. *Pediatr. Res.* **94**, 1317–1326 (2023). 10.1038/s41390-023-02610-5 PMC10589081 37138028

[28] Guardado, M. et al. The urinary metabolomic fingerprint in extremely preterm infants on total parenteral nutrition vs. enteral feeds. *Metabolites* **13**, 971 (2023). 10.3390/metabo13090971 PMC10537655 37755251

[29] Abdel-Hady, H., Shouman, B. & Aly, H. Early weaning from CPAP to high flow nasal cannula in preterm infants is associated with prolonged oxygen requirement: a randomized controlled trial. *Early Hum. Dev.* **87**, 205–208 (2011). 10.1016/j.earlhumdev.2010.12.010 21276671

[30] Todd, D. A. et al. Methods of weaning preterm babies <30 weeks gestation off CPAP: a multicentre randomised controlled trial. *Arch. Dis. Child Fetal Neonatal Ed.* **97**, F236–F240 (2012). 10.1136/adc.2011-300133 22611116

[31] McEvoy, C., Bowling, S., Williamson, K., Stewart, M. & Durand, M. Functional residual capacity and passive compliance measurements after antenatal steroid therapy in preterm infants. *Pediatr. Pulmonol.* **31**, 425–430 (2001). 10.1002/ppul.1070 11389574

[32] McEvoy, C. T. et al. Vitamin C supplementation for pregnant smoking women and pulmonary function in their newborn infants: a randomized clinical trial. *JAMA* **311**, 2074–2082 (2014). 10.1001/jama.2014.5217 PMC4296045 24838476

[33] Morris, M. G. et al. The bias flow nitrogen washout technique for measuring the functional residual capacity in infants. *ERS/ATS Task. Force Stand. Infant Respir. Funct. Test. Eur. Respir. J.* **17**, 529–536 (2001). 10.1183/09031936.01.17305290 11405534

[34] Castillo, A. et al. Measurement of single breath-hold carbon monoxide diffusing capacity in healthy infants and toddlers. *Pediatr. Pulmonol.* **41**, 544–550 (2006). 10.1002/ppul.20403 16617450

[35] Praca, E. L. L., Tiller, C. J., Kisling, J. A. & Tepper, R. S. An alternative method to measure the diffusing capacity of the lung for carbon monoxide in infants. *Pediatr. Pulmonol.* **53**, 332–336 (2018). 10.1002/ppul.23926 29265767

[36] Jones, M. et al. Forced expiratory flows and volumes in infants: normative data and lung growth. *Am. J. Respir. Crit. Care Med.* **161**, 353–359 (2000). 10.1164/ajrccm.161.2.9903026 10673171

[37] Rings, E. H. et al. Functional development of fat absorption in term and preterm neonates strongly correlates with ability to absorb long-chain Fatty acids from intestinal lumen. *Pediatr. Res.* **51**, 57–63 (2002). 10.1203/00006450-200201000-00011 11756640

[38] Reinke, S. N., Chaleckis, R. & Wheelock, C. E. Metabolomics in pulmonary medicine: extracting the most from your data. *Eur. Respir. J.* **60**, 2200102 (2022). 10.1183/13993003.00102-2022 PMC9386331 35618271

[39] Wittmer, V. L., Simoes, G. M., Sogame, L. C. & Vasquez, E. C. Effects of continuous positive airway pressure on pulmonary function and exercise tolerance in patients with congestive heart failure. *Chest* **130**, 157–163 (2006). 10.1378/chest.130.1.157 16840396

[40] Mansfield, D. & Naughton, M. T. Effects of continuous positive airway pressure on lung function in patients with chronic obstructive pulmonary disease and sleep disordered breathing. *Respirology* **4**, 365–370 (1999). 10.1046/j.1440-1843.1999.00206.x 10612570

[41] Gerhardt, T., Reifenberg, L., Hehre, D., Feller, R. & Bancalari, E. Functional residual capacity in normal neonates and children up to 5 years of age determined by a N2 washout method. *Pediatr. Res.* **20**, 668–671 (1986). 10.1203/00006450-198607000-00018 3725466

[42] Wang, G. & Rao, H. Effective CPAP Treatment is Associated with Catch Up Growth in Infants with Obstructive Sleep Apnea and Failure to Thrive. *Sleep* **41**, A419 (2018).

[43] Siopi, D. & Steiropoulos, P. The influence of CPAP therapy on basal metabolic rate and physical activity in obese patients with obstructive sleep apnea. *Nutrients* **15**, 4446 (2023). 10.3390/nu15204446 PMC10609663 37892521

[44] Tachikawa, R. et al. Changes in energy metabolism after continuous positive airway pressure for obstructive sleep apnea. *Am. J. Respir. Crit. Care Med.* **194**, 729–738 (2016). 10.1164/rccm.201511-2314OC 26930227

[45] Saito, K. et al. Gender- and age-associated differences in serum metabolite profiles among Japanese populations. *Biol. Pharm. Bull.* **39**, 1179–1186 (2016). 10.1248/bpb.b16-00226 27374292

[46] Takayasu, T. et al. Cerebrospinal fluid ctDNA and metabolites are informative biomarkers for the evaluation of CNS germ cell tumors. *Sci. Rep.* **10**, 14326 (2020). 10.1038/s41598-020-71161-0 PMC7459305 32868820

[47] Fang, J. et al. Dihydro-orotate dehydrogenase is physically associated with the respiratory complex and its loss leads to mitochondrial dysfunction. *Biosci. Rep.* **33**, e00021 (2013). 10.1042/BSR20120097 PMC3564035 23216091

[48] Martin, C. R. et al. Decreased postnatal docosahexaenoic and arachidonic acid blood levels in premature infants are associated with neonatal morbidities. *J. Pediatr.* **159**, 743–749 e741-742 (2011). 10.1016/j.jpeds.2011.04.039 PMC3701520 21658712

[49] Van Hove, J. L. et al. Medium-chain acyl-CoA dehydrogenase (MCAD) deficiency: diagnosis by acylcarnitine analysis in blood. *Am. J. Hum. Genet.* **52**, 958–966 (1993). PMC1682046 8488845

[50] Vissing, C. R., Duno, M., Wibrand, F., Christensen, M. & Vissing, J. Hydroxylated long-chain acylcarnitines are biomarkers of mitochondrial myopathy. *J. Clin. Endocrinol. Metab.* **104**, 5968–5976 (2019). 10.1210/jc.2019-00721 31294795

[51] Semba, R. D. et al. Environmental enteric dysfunction is associated with carnitine deficiency and altered fatty acid oxidation. *EBioMedicine* **17**, 57–66 (2017). 10.1016/j.ebiom.2017.01.026 PMC5360565 28122695

[52] Langley, R. J. et al. A metabolomic endotype of bioenergetic dysfunction predicts mortality in critically ill patients with acute respiratory failure. *Sci. Rep.* **11**, 10515 (2021). 10.1038/s41598-021-89716-0 PMC8131588 34006901

[53] Shen, B. et al. Proteomic and metabolomic characterization of COVID-19 patient sera. *Cell* **182**, 59–72 e15 (2020). 10.1016/j.cell.2020.05.032 PMC7254001 32492406

[54] Migaud, M. et al. Metabolomics to predict antiviral drug efficacy in COVID-19. *Am. J. Respir. Cell Mol. Biol.* **63**, 396–398 (2020). 10.1165/rcmb.2020-0206LE PMC7462337 32574504

[55] Sindelar, M. et al. Longitudinal metabolomics of human plasma reveals prognostic markers of COVID-19 disease severity. *Cell Rep. Med.* **2**, 100369 (2021). 10.1016/j.xcrm.2021.100369 PMC8292035 34308390

[56] Fraser, D. D. et al. Metabolomics profiling of critically ill coronavirus disease 2019 patients: identification of diagnostic and prognostic biomarkers. *Crit. Care Explor* **2**, e0272 (2020). 10.1097/CCE.0000000000000272 PMC7587450 33134953

[57] Elshenawy, S. et al. The metabolomic signature of the placenta in spontaneous preterm birth. *Int. J. Mol. Sci.* **21** (2020). 10.3390/ijms21031043 PMC7037776 32033212

[58] Zong, Y. et al. Mitochondrial dysfunction: mechanisms and advances in therapy. *Sig. Transduct. Targeted Therapy* **9** (2024). 10.1038/s41392-024-01839-8 PMC11094169 38744846

[59] Vitek, L., Zelenka, J., Zadinova, M. & Malina, J. The impact of intestinal microflora on serum bilirubin levels. *J. Hepatol.* **42**, 238–243 (2005). 10.1016/j.jhep.2004.10.012 15664250

[60] Al-Hosni, M. et al. Probiotics-supplemented feeding in extremely low-birth-weight infants. *J. Perinatol.* **32**, 253–259 (2012). 10.1038/jp.2011.51 21546942

[61] Angelakis, E. & Raoult, D. Gut microbiota modifications and weight gain in early life. *Hum. Microbiome J.* **7**, 10–14 (2018).

[62] Kong, S. W. & Hernandez-Ferrer, C. Assessment of coverage for endogenous metabolites and exogenous chemical compounds using an untargeted metabolomics platform. *Pac. Symp. Biocomput.* **25**, 587–598 (2020). PMC6910716 31797630

[63] Martin, R. J., Wang, K., Koroglu, O., Di Fiore, J. & Kc, P. Intermittent hypoxic episodes in preterm infants: do they matter? *Neonatology* **100**, 303–310 (2011). 10.1159/000329922 PMC3252018 21986336

[64] Jensen, E. A. et al. Association between intermittent hypoxemia and severe bronchopulmonary dysplasia in preterm infants. *Am. J. Respir. Crit. Care Med.* **204**, 1192–1199 (2021). 10.1164/rccm.202105-1150OC PMC8759313 34428130

[65] Salas, A. A. et al. Growth rates of infants randomized to continuous positive airway pressure or intubation after extremely preterm birth. *J. Pediatr.* **237**, 148–153 e143 (2021). 10.1016/j.jpeds.2021.06.026 PMC8478787 34157349

[66] Di Fiore, J. M., MacFarlane, P. M. & Martin, R. J. Intermittent hypoxemia in preterm infants. *Clin. Perinatol.* **46**, 553–565 (2019). 10.1016/j.clp.2019.05.006 PMC6662623 31345546

[67] Darnall, R. A. et al. Early postnatal exposure to intermittent hypoxia in rodents is proinflammatory, impairs white matter integrity, and alters brain metabolism. *Pediatr. Res.* **82**, 164–172 (2017). 10.1038/pr.2017.102 PMC5509485 28388601

[68] Thompson, L., Werthammer, J. W. & Gozal, D. Apnea of prematurity and oxidative stress: potential implications. *Antioxidants* **13** (2024). 10.3390/antiox13111304 PMC11591010 39594446

[69] Nanduri, J. et al. Intermittent hypoxia degrades HIF-2alpha via calpains resulting in oxidative stress: implications for recurrent apnea-induced morbidities. *Proc. Natl. Acad. Sci. USA* **106**, 1199–1204 (2009). 10.1073/pnas.0811018106 PMC2626608 19147445

[70] Yuan, G., Nanduri, J., Khan, S., Semenza, G. L. & Prabhakar, N. R. Induction of HIF-1alpha expression by intermittent hypoxia: involvement of NADPH oxidase, Ca2+ signaling, prolyl hydroxylases, and mTOR. *J. Cell Physiol.* **217**, 674–685 (2008). 10.1002/jcp.21537 PMC2696817 18651560

[71] Mamidi, R. H. et al Stable Preterm infants randomized to extended continuous positive airway pressure (eCPAP) versus discontinuation of CPAP (dCPAP) in the NICU have decreased episodes of intermittent hypoxemia (IH) *Pediatric Academic Societies Meeting* (2025).

[72] Yazdi, S. et al. Extended CPAP or low-flow nasal cannula for intermittent hypoxaemia in preterm infants: a 24-hour randomised clinical trial. *Arch. Dis. Child Fetal Neonatal Ed.* **109**, 557–561 (2024). 10.1136/archdischild-2023-326605 PMC11327380 38365446

