# Supplementary material for: Metabolic response to extended continuous positive airway pressure in premature infants
Source: Pediatr Res. 2025 Dec 22;100(2):885–95. doi: 10.1038/s41390-025-04670-1 (PMC13638474; doi:10.1038/s41390-025-04670-1)
Supplement: Supplementary file 1 [file 41390_2025_4670_MOESM1_ESM.pdf]

Supplemental Figure S1

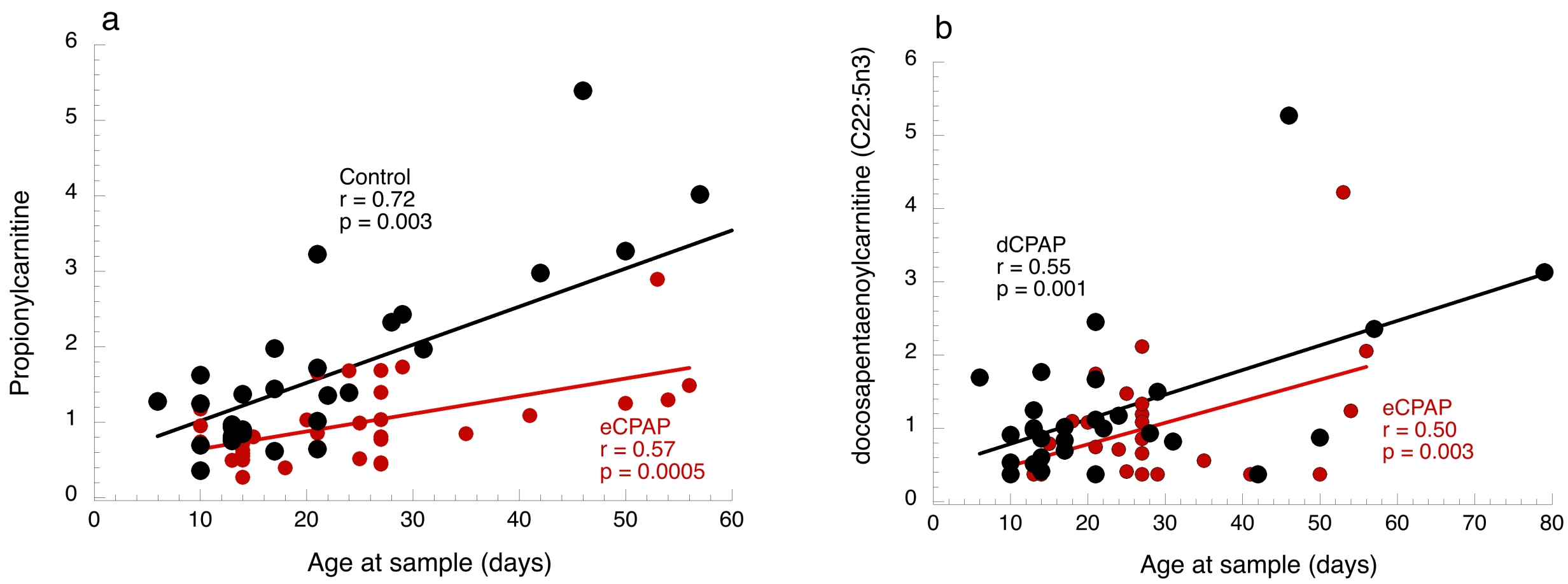

Supplemental Figure S1. Levels of two acylcarnitines by postnatal age at collection of blood sample. a, propionylcarnitine (C3); b) docosapentaenoylcarnitine (C22). Analysis by linear regression: eCPAP n=33, dCPAP n=31.
